# Perilla Oil and *Bifidobacterium* for Alleviating Fear of Cancer Recurrence in Breast Cancer Survivors: Study Protocol for a Three-Arm Phase II Randomized Controlled Study (POB Study)

**DOI:** 10.3390/mps4030046

**Published:** 2021-07-06

**Authors:** Yohei Sasaki, Mina Honyashiki, Takayuki Kinoshita, Akira Matsui, Ayako Nakashoji, Takuma Inagawa, Satoru Ikezawa, Naoki Yoshimura, Ryodai Yamamura, Mizuki Amano, Yui Tomo, Hisateru Tachimori, Yutaka J. Matsuoka, Ryo Okubo

**Affiliations:** 1 Department of Clinical Epidemiology, Translational Medical Center, National Center of Neurology and Psychiatry, Tokyo 187-8551, Japan; ysasaki@ncnp.go.jp (Y.S.); tomo-yui031@g.ecc.u-tokyo.ac.jp (Y.T.); tachi@ncnp.go.jp (H.T.); 2 Department of Psychiatry, Tokyo Medical University, Tokyo 113-8510, Japan; honyashiki@outlook.jp; 3 Department of Breast Surgery, National Hospital Organization Tokyo Medical Center, Tokyo 187-8551, Japan; kinoshita.takayuki.yg@mail.hosp.go.jp (T.K.); matsui.akira.fa@mail.hosp.go.jp (A.M.); nakashoji.ayako.re@mail.hosp.go.jp (A.N.); 4 Department of Psychiatry, National Center Hospital of Neurology and Psychiatry, Tokyo 187-8551, Japan; takumainagawa@gmail.com (T.I.); satoru.ikezawa@ncnp.go.jp (S.I.); naoyoshi@ncnp.go.jp (N.Y.); 5 Division of Biomedical Oncology, Institute for Genetic Medicine, Hokkaido University, Sapporo 060-0815, Japan; ryamamura@igm.hokudai.ac.jp; 6 Toyosato Hospital, Ibaraki 529-1168, Japan; amano2951@gmail.com; 7 Division of Health Care Research, Center for Public Health Sciences, National Cancer Center Japan, Tokyo 104-0045, Japan; matsuoka-psy@umin.ac.jp; 8 Lifestyle Medicine, Cooperative Graduate Program, The Jikei University Graduate School of Medicine, Tokyo 105-8461, Japan

**Keywords:** fear of cancer recurrence, breast cancer, microbiome, probiotics, omega-3 fatty acids

## Abstract

The fear of cancer recurrence (FCR) is the most common and most severe unmet need among cancer survivors. Safe treatments for the FCR that are easily disseminated are greatly needed. Our primary aim is a preliminary evaluation of the efficacy and effect size of perilla oil, which is rich in omega-3 fatty acids, and *Bifidobacterium*, a probiotic, on FCR in breast cancer survivors after the completion of chemotherapy. This study has been planned as an exploratory clinical study (phase II) and will be conducted as a three-arm, 12-week parallel group, masked-rater randomized controlled trial. Fifteen participants will be randomized with 1:1:1 allocation to receive *Bifidobacterium* plus perilla oil, *Bifidobacterium* alone, or no intervention (control). Interventions will end within 12 weeks after the random allocation of each participant. The participants will be outpatients with invasive breast cancer aged 20 years or older whose chemotherapy was completed at least 6 months before registration; hormone therapy may be ongoing. The primary outcome will be severity of FCR at 12 weeks assessed by masked raters using the 4-item Concerns about Recurrence Scale concerning overall fear of recurrence. The study protocol for the current study is registered in the Japan Registry of Clinical Trials (jRCTs031200029).

## 1. Introduction

Among various types of psychological distress experienced by cancer survivors, the fear of cancer recurrence (FCR) is the most common and the most severe problem for which adequate support is often not available [1]. Longitudinal studies have shown that FCR can persist for years after finishing initial treatment, with more than half of cancer survivors experiencing moderately severe FCR and 7% experiencing extremely severe FCR [1]. Additionally, FCR is associated with after-hour visits, reduced quality of life, and reduced social functioning [1], suggesting the need for appropriate management. With the exception of extremely severe FCR, for which pharmacological and/or psychiatric therapies are implemented in line with current treatment practices for anxiety disorder, FCR is generally considered to be an extension of normal psychology and was reported to be the most frequently endorsed unmet need at the end of initial treatment and 6 months post-treatment in a multicenter study conducted in the United Kingdom [2]. A Japanese study of 408 breast cancer survivors also reported that 63% sought FCR-related care during treatment [3]. There is an ongoing need, therefore, for safe treatments for FCR that can be easily disseminated and that can offer cancer survivors a sense of security.

In previous work, psychological models were used to develop psychological therapies for FCR [4,5,6,7,8]. Additionally, it has also been suggested that there is an association between FCR and various biological risk factors, such as the presence and severity of physical symptoms and a history of chemical or radiation therapies [9]. FCR is a phenomenon in which reminders of cancer trigger the negative interpretation and recollection of fear memory, [4] and the degree of FCR is strongly related to intrusive memories as a symptom of post-traumatic stress disorder (PTSD) [10]. The pathology of PTSD is explained as the abnormal processing of fear memory, such as the enhancement of trauma-related fear memory consolidation [11] and the impairment of fear memory extinction [12]. This suggests that the dysregulation of fear memories is involved in the underlying cause of FCR.

Extensive studies investigating the regulation of fear memories have revealed that n-3 polyunsaturated fatty acids (PUFAs) [13,14,15] and gut microbiota play important roles in the brain functions related to fear memory regulation [16,17]. Our recent review suggested that n-3 PUFAs and the modulation of the microbiota–gut–brain axis improve dysfunctional fear memory processing via immunomodulation/anti-inflammation, increased levels of the brain-derived neurotrophic factor, upregulated adult neurogenesis, and modulated signal transduction [1]. Based on this, we hypothesized that an association exists among FCR, omega-3 PUFAs, and intestinal flora, and we conducted an observational study in 130 breast cancer survivors. We found lower FCR in those with higher blood levels of alpha-linolenic acid (ALA; an omega-3 PUFA found in perilla oil, linseed oil, and walnuts), and the association was independent of other psychiatric symptoms such as depressive symptoms [18]. We also found that FCR showed a positive correlation with the burden of Bacteroides, a proinflammatory bacterial species, and a negative correlation with the abundance of Firmicutes bacteria, which mainly produce butyric acid, a short-chain FA involved in the maintenance of the intestinal environment by improving intestinal barrier function. There was also a negative correlation with the diversity of the overall intestinal bacteria and a particularly strong correlation with the intestinal environment in patients after they had completed chemotherapy [19]. Based on these observations, we conceptualized the present study in which we will examine the effectiveness of nutritional intervention for cancer survivors with FCR.

Probiotics are live microorganisms that confer health benefit on the host when administered in adequate amounts (FAO/WHO Joint Working Group, 2001). Numerous studies have reported improved symptoms in patients with psychiatric disorders following probiotic interventions [20,21,22,23,24,25], and there is growing evidence of an interaction between omega-3 PUFAs and gut microbiota [26,27,28,29]. Diets rich in fish oil, which includes omega-3 PUFAs, increase *Bifidobacterium* levels and decrease Bacteroid levels compared to a diet rich in corn and beef fat [30]. Fat-1 transgenic mice engineered with the *Caenorhabditis elegans* Fat-1 gene can produce omega-3 PUFAs from Omega-6 PUFAs in their organs and tissues. The Fat-1 transgenic mice showed more production and secretion of intestinal alkaline phosphatase (IAP) than mice fed omega-6 PUFAs; the IAP decreased LPS production and gut permeability and suppressed endotoxemia and inflammation [31]. However, no studies have examined the effects of the combination of probiotics and omega-3 PUFAs to date.

This pilot study primarily aims to evaluate the efficacy and effect size of taking perilla oil rich in omega-3 PUFAs and *Bifidobacterium* as a probiotic on FCR in breast cancer survivors after they have completed chemotherapy. We believe the preliminary results will help to guide future full-scale confirmatory trials on their effects. Given the growing interest in the interaction between omega-3 PUFAs and intestinal flora, this study will also explore the optimal dosing regimen (*Bifidobacterium* alone vs. *Bifidobacterium* plus perilla oil).

## 2. Methods and Analysis

### 2.1. Study Design

This study has been planned as an exploratory clinical study (phase 2) and will be conducted as a three-arm, 12-week parallel group, masked-rater randomized controlled trial (RCT). The study is registered in the Japan Registry of Clinical Trials (jRCT; Trial ID jRCTs031200029). Participants will be randomized with 1:1:1 allocation to receive Bifidobacterium plus perilla oil, Bifidobacterium alone, or no intervention (control). A total of 15 participants will be allocated to each arm. These interventions will end within 12 weeks of the registration of each participant. The primary outcome will be FCR severity at 12 weeks as assessed by masked raters using the 4-item Concerns about Recurrence Scale (CARS) that asks about overall fear of recurrence.

### 2.2. Inclusion Criteria

1. Aged 20 years or older at the time of providing informed consent;
2. Outpatient at the National Center of Neurology and Psychiatry hospital or the National Hospital Organization Tokyo Medical Center;
3. Histologically or cytologically confirmed invasive breast cancer without cancer recurrence. The criteria is limited to invasive breast cancers because they are more likely to recur than non-invasive breast cancers and tend to be associated with a higher level of (more severe) FCR;
4. Chemotherapy completed at least 6 months before registration. Hormone therapy may be ongoing. Patients who have only just completed chemotherapy will not be included because their diets tend to change due to stomatitis, and their gut environment can be unstable due to acute intestinal inflammation from the chemotherapy;
5. At least a moderate level of fear (scoring ≥ 3 points) for the item of intensity of fear on the CARS, as previously reported by studies using the CARS [32,33];
6. Recognition of cancer diagnosis. This criterion is set because a small number of people do not recognize themselves as having cancer, but the patients in the present study must recognize themselves as cancer survivors.

### 2.3. Exclusion Criteria

1. Severe cognitive impairment due to delirium, dementia, or other problems. Patients with severe cognitive impairment are excluded since they might be unable to properly consume the test food or respond to questions independently;
2. No Japanese literacy;
3. Ongoing non-hormonal anticancer therapy for breast or other cancers;
4. Need for immediate psychiatric intervention for suicidal ideation or other severe psychiatric symptoms at study entry;
5. No antianxiety or antidepressant medication added or discontinued in the preceding month;
6. Daily habit of consuming any ALA-containing food (e.g., perilla or linseed oil). That is, we will exclude patients who are intentionally consuming ALA-containing food daily;
7. Daily habit of consuming Bifidobacterium-containing food. These patients are excluded to prevent over- or underestimation of the effect of the intervention. That is, we will exclude patients who are intentionally consuming Bifidobacterium-containing food daily;
8. Deemed by the attending physician to be unfit for inclusion in the study for any other reasons. For this criterion, it is necessary to consider the possibility of extreme bias in the patients to be excluded due to the personal opinion of individual physicians. Therefore, the reason for exclusion should be documented whenever this criterion is met.

### 2.4. Participant Timeline and Recruitment

**Figure 1:**
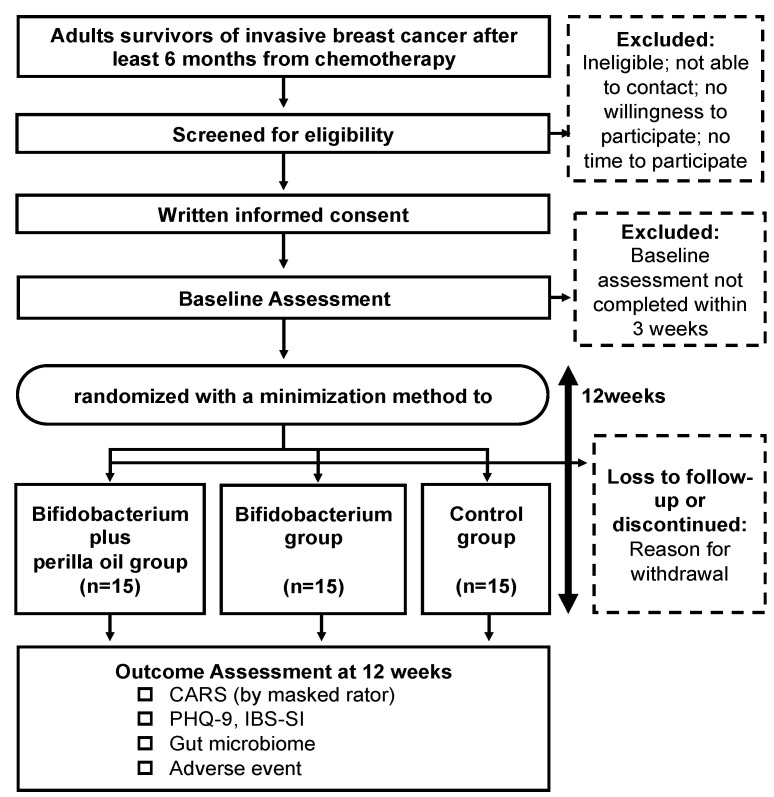
Flowchart of this trial.

Patients will be recruited at the National Center for Neurology and Psychiatry (NCNP) hospital and National Hospital Organization (NHO) Tokyo Medical Center in Tokyo, Japan. Posters with study information will be displayed on the walls of the outpatient waiting room in both hospitals. Eligible patients will be screened through their medical records by their physician-in-charge. After the screening, all patients who meet the eligibility criteria will be approached by the research team in order to confirm the criteria. Those who satisfy all inclusion criteria and none of the exclusion criteria will receive a brief explanation of the study from the physician-in-charge. The clinical research coordinator (CRC) will provide sufficient written and verbal explanations and obtain written informed consent from the participants. Participants will then undergo baseline assessments. Figure 1 shows the flow of participants through the study.

### 2.5. Randomization and Masking

After completing the baseline assessment, participants will be randomly assigned at a of 1:1:1 allocation ratio to one of the three groups: the Bifidobacterium plus perilla oil group, the Bifidobacterium group, or the control group. Within the Hope eACReSS electronic data capture system (EDC) system, made available by the University Hospital Clinical Trail Alliance (http://plaza.umin.ac.jp/~UHCTA/edc.html, accessed on 5 July 2021), randomization will be performed using a minimization method and will be balanced using the following two factors: stratification age (<65 years, ≥65 years) and duration from diagnosis (<5 years, ≥5 years).

The primary outcome will be evaluated by the CRC and masked raters. At baseline, the CRC will evaluate the outcome before allocation. At 12 weeks, raters masked to all information regarding the allocation of this study will evaluate this outcome over the phone. The primary investigator (RO) will hold a workshop on the primary outcome evaluation for the CRC and masked raters using simulated recordings to confirm the evaluation method and will assess the inter-rater reliability for score evaluation. The primary outcome, FCR, is a subjective concept and result, and the CARS used in the evaluation is a self-reported scale, but in order to standardize the conditions of the responses, a masked rater will read the questionnaire aloud and will spend 5 min conducting the evaluation. This procedure is based on previous studies in which the primary outcome was a self-reported measure evaluated by masked raters [34,35].

### 2.6. Intervention Groups

In this three-arm RCT, two regimens of nutritional intervention, Bifidobacterium plus perilla oil and Bifidobacterium alone, will be compared to the control group. These two interventions will be completed within 12 weeks of the random allocation of the interventions. We have chosen perilla oil, which is rich in ALA, as an omega-3 supplement based on our observational and other studies. Consumption of a diet rich in ALA facilitates the inhibitory learning of original fear memories more than a diet rich in linolenic acids, one of the n-6 PUFAs, in rats [36]. An ALA-rich chocolate milkshake was recently found to facilitate the inhibitory learning of original fear memories in healthy individuals [37].

Participants in the Bifidobacterium plus perilla oil group will be asked to take 2 capsules (10 billion Bifidobacterium bacteria per capsule) and 1 sachet containing 3 g perilla oil (1.8 g ALA) daily with meals. Perilla oil may be mixed with soup or poured over salad or yogurt. Participants in the Bifidobacterium group will be asked to take 2 capsules (10 billion Bifidobacterium bacteria per capsule) with breakfast. Although there will be no restriction on concomitant drugs/treatments/procedures, information regarding concomitant drugs/therapies during the study will be collected as study data.

To enhance treatment adherence, participants will be contacted by telephone or email at 0, 4, 8, and 12 weeks of the study period. Participants will be informed that if they forget to take a capsule or sachet, they can take a 2-day dose at one time. They will be instructed not to chew the Bifidobacterium capsules. Participants in the intervention groups will be asked to report the number of weeks (0–12) that they have been taking their capsules and sachets during the 12 weeks and the reasons why they did not take them if they took ≤7 (i.e., compliance rate <60%).

### 2.7. Criteria for Discontinuing Allocated Interventions

The intervention protocol will be discontinued for any of the following reasons: (1) drop-out due to withdrawal of consent or declining to participate; (2) severe side effects (e.g., significant diarrhea); (3) sudden worsening of the participant’s condition after enrolment; (4) loss of contact with the participant; (5) the Principal Investigator and co-researchers determine that the mental and physical burden of the study on the participant is too much to continue the research(e.g., when life events or complications prevent the patient from consuming test foods, answering questionnaires, or submitting stool samples); and (6) deviation from the research protocol, such as when the selection criteria and exclusion criteria are found to be violated after taking the test capsules and sachets. 

### 2.8. Control Group

There will be no placebo capsule or sachet in this phase II trial. The reasons for this are detailed in Section 4. Participants in the control group will not receive any intervention during the study period. Although there will be no restriction on concomitant drugs/treatments/procedures, information regarding concomitant drugs/therapies used during the study will be collected as study data. Whenever inquiries are received from participants, the necessary information will be provided to them.

### 2.9. Data Collection Methods

The following information will be collected at the baseline assessment: age, sex, marital status, cancer stage, surgical history, history of radiation therapy, work status, family status, medication history (mental illness, other disease, antibiotic use, psychotropic drugs), body height/weight, and diet assessed using the Food Frequency Questionnaire (FFQ) [38,39]. For baseline characteristics, information will be collected from medical records as well as from the individual participants through questionnaires and interviews. We will also assess blood fatty acids at baseline with a custom test kit (Xerion Pharmaceuticals, Victoria, Australia) using dried blood spot testing as described previously [19]. All participants will receive a gift voucher for 2000 JPY (approximately 18 USD) for their participation after each assessment. An overview of the schedule for the assessments is shown in Table 1.

### 2.10. Primary Outcome Measures

The primary outcome will be FCR severity at 12 weeks as assessed by the masked raters using the 4-item CARS. The CARS comprises 30 questions [33] of which 4 concern overall fear of recurrence; the remaining 26 questions measure worries associated with recurrence under specific circumstances such as “worries about female sexuality” and “worries about future treatment”. These 26 questions will not be asked in this study. The 4 questions regarding overall fear of recurrence address the frequency, potential for upset, consistency, and intensity of fear. Each question is scored from 1 (no fear) to 6 (intense/frequent fear), giving an average score of 1 to 6, with higher scores indicating greater fear of recurrence. The validity of the Japanese version has been confirmed [40]. Participants who score ≥3 on overall fear of CARS will be categorized as having a moderate or high level of FCR [32,33].

### 2.11. Secondary Outcome Measures

Depressive symptoms will be assessed using the Japanese version of the Patient Health Questionnaire-9 (PHQ-9) [41]. The PHQ-9 is a self-rated questionnaire and focuses on 9 items regarding DSM-5 defined major depression in a structured diagnostic interview called the Primary Care Evaluation of Mental Disorders. Focusing on the preceding 2 weeks, each question is scored from 0 to 3, giving a total score of 0 to 27, where a lower the score was associated with a less depressed mood.

Irritable bowel syndrome (IBS) will be assessed using the Japanese version of the IBS severity index (IBS-SI) [42]. Total scores range from 0 to 500, summed from four subjective aspects; abdominal pain, abdominal distension, bowel movement, and quality of life. IBS is graded as mild (75–174), moderate (175–299), or severe (300–500) based on clinical observations of IBS patients [43].

Gut microbiota will be assessed using the participants’ fecal samples, which will be collected using a custom test kit and analyzed using meta-16S rRNA analysis as described previously [19].

### 2.12. Harm

Adverse reactions (ARs) will be collated as secondary outcomes. At 12 weeks after random allocation, we will specifically ask if participants have had any of the following ARs: headache, nausea, diarrhea, loose stool, and burping (if any, we will also ask about the time of onset, severity, subjective severity, need for treatment, and prognosis). The ARs of the participants in the intervention group will be monitored over the telephone by the CRC at 4 and 8 weeks after entry into the study. This step is based on previous studies reporting an 18% increased risk of ARs with omega 3 supplements compared to a placebo. The most common ARs were gastrointestinal and included nausea, diarrhea, and other minor gastrointestinal disturbances [44]. Clinical trials using Bifidobacterium have been conducted with only small sample sizes, and the ARs remain unclear.

An Efficacy and Safety Committee will be established, comprising an independent chair and at least two independent members. In the event of a serious illness for which a causal relationship to the intervention cannot be ruled out, this Committee will convene. If three serious illnesses for which a causal relationship to the intervention cannot be ruled out occur, the study will be stopped immediately without waiting for review by the Committee. The treatment of and care for ARs in patients enrolled in the study are covered by their medical insurance.

Participants who experience a substantial worsening of FCR or depressive symptoms during the study period will be promptly referred to a psychiatrist in collaboration with their physician. All of the test foods used in this study are commercially available and are well-established dietary products. Therefore, this study will not be covered by clinical research insurance. In the unlikely event that a research subject suffers health problems as a result of participation in this study, appropriate measures will be taken to provide the necessary treatment, including examination and treatment, within the research subject’s insurance coverage.

### 2.13. Sample Size Calculation

The sample size was estimated based on power analysis using the CARS score. To our knowledge, no previous reports have examined the effects of nutritional interventions on FCR, so there is no reference on which we can base our power calculation.

In our observational study conducted between 2017 and 2018, the mean CARS score of a subset of breast cancer survivors who met the eligibility criteria for this study with a prior history of chemotherapy and CARS score ≥3 was 3.96 (SD ± 0.67). Furthermore, in this study, the intervention will be considered clinically significant if the mean CARS score at week 12 in the intervention group is 1 point lower than the mean CARS score at week 12 in the control group. The CARS score ranges from 1 (not worried at all) to 6 (always very worried). In the opinion of breast surgery specialists (TK and AM), a 1-point decrease in this score from 4 (somewhat worried) to 3 (slightly worried) is clinically significant.

Thus, based on the above hypothesis and the results of our previous study, the mean difference in CARS scores was estimated to be 1.0 ± 0.67 (SD) between the Bifidobacterium plus perilla oil group and the control group and between the Bifidobacterium and the control groups at 12 weeks after random allocation. To detect the difference between the two groups, using unpaired *t*-tests with a one-sided alpha of 2.5% and power of 90%, the calculation showed that 11 participants per group would be required in the analysis population. This was calculated using G*Power software (ver.3.1.9.4). Assuming 4 dropouts per group, 15 participants will be enrolled per group (45 in total).

### 2.14. Statistical Methods

This study is a phase II trial primarily focused on the effects of Bifidobacterium and perilla oil when taken properly. Therefore, the main analysis will be performed in a per protocol set (PPS), which is defined as participants who complete the final post-intervention primary outcome assessment and complete the protocol-specified interventions. Completion of the protocol-specified interventions is defined as completion of least 4 of the 12 weeks of Bifidobacterium and perilla oil administration; these participants will be considered to have completed the protocol-specified interventions based on a meta-analysis of probiotic interventions for anxiety symptoms where the minimum treatment period in studies demonstrating efficacy was 4 weeks [45].

In the secondary analysis, analysis will be performed on all randomized cases based on the intention-to-treat (ITT) principle, and discrepancies in the analysis using the PPS will also be examined. Regarding patient background, summary statistics (mean, standard deviation, and frequency) will be calculated based on the characteristics of the items, and the presence or absence of bias in the three baseline groups in each item will be evaluated based on analysis of variance or the chi-squared test.

The objective of the primary analysis of this study is to collect data to inform a main confirmatory clinical trial (phase III). Regarding the primary outcome, the primary hypothesis is that the mean CARS scores of the intervention groups (Bifidobacterium alone or Bifidobacterium plus perilla oil) at week 12 will be significantly lower than those of the control group. The intervention will be considered effective if the null hypothesis is rejected. The difference in the mean CARS score at 12 weeks after random allocation between the intervention groups and the control group will be analyzed using the unpaired t-test. In the primary analysis, we will not compare the effects between the two intervention groups. Missing values or modeling will not be imputed for primary analysis. Sensitivity analyses will be performed for the primary outcome after imputing missing data using the multiple imputation method.

The following secondary and sensitivity analyses will be performed for additional discussion of the primary analysis, which will determine the need for further studies. These analyses will be conducted after the primary analysis is completed. For all continuous outcomes (CARS, PHQ-9, IBS-SI, and gut microbiome composition), differences in the scores at 12 weeks and changes between baseline and 12 weeks will be examined using non-paired t-tests and mixed-effect models for repeated measures analysis using repeated timepoints and age, disease duration, and hospital as covariates. For dichotomous or dichotomized categorical outcomes such as ARs and proportion of participants with a moderate or high level of FCR at 12 weeks, relative risk will be calculated. Chi-squared and Fisher’s exact tests will be used for statistical significance testing. The aforementioned analyses will be performed between the combined intervention group (those treated with Bifidobacterium alone and those treated with Bifidobacterium plus perilla oil; *n* = 30) and the control group (*n* = 15). The purpose of this analysis is to increase the power of the hypothesis test to examine whether Bifidobacterium intervention is effective against FCR. This is a supplemental analysis to reinforce the arguments of the primary analysis. Furthermore, to explore the association of changes in symptoms with the gut microbiome and PUFAs, we will perform correlation analyses.

This study will not include an interim analysis. If either of the intervention groups (Bifidobacterium alone and Bifidobacterium plus perilla oil) are inferior to the usual treatment group (control group), it is not a matter of concern whether it is statistically significant or not, so the test will be one-sided. The significance level of the entire test is 2.5% for one side. While this decision might lead to errors in multiple tests, this will be the first study of nutritional intervention for FCR. Therefore, we consider the avoidance of beta errors to be preferable over alpha errors. However, 95% confidence intervals will be presented for differences among groups.

### 2.15. Data Management and Monitoring and Auditing

Before the study commences, we will fix the data management process including the case report form (CRF) and will use the EDC system. We have appointed Kenji Hatano, M.D., who is an expert on data management and monitoring, as the Director of Data Management and Monitoring. To ensure that the quality of the data is in accordance with the predetermined protocol, data monitoring will be conducted. Under the supervision of Dr. Hatano, an independent monitor will examine whether the recruitment procedure and data recording adhere to the protocol in the CRF.

This study will be conducted for exploratory research purposes, using commercially available foods with sufficient dietary experience and that pose minimal risk and minor invasiveness. Therefore, data monitoring and auditing by a data monitoring committee are not considered mandatory for this trial. However, in the case that any of the following occur, auditing will be conducted: (1) two serious illnesses for which a causal relationship to the intervention cannot be ruled out; (2) two cases of serious deviation from the protocol; and (3) incidents of critical conflicts of interests.

## 3. Ethics and Dissemination

### 3.1. Ethical Considerations

This study complies with the principles of the Declaration of Helsinki and Clinical Trials Act (No.16 of 14 April 2017) in Japan. This study protocol was approved by the National Center of Neurology and Psychiatry Clinical Research Review Board (CRB 3180006) on 1 Januray 2020 (approval number: CR19-003). This study protocol paper is based on the ver.1.7 protocol (amended approval obtained on 5 February 2020). For any important protocol modifications such as changes to the eligibility criteria, outcomes, or analyses, a formal amendment to the protocol is required.

Written informed consent will be obtained from all participants by the physicians-in-charge and CRC, ensuring sufficient written and verbal explanations. Participants will be informed that they can withdraw from the study at any time without any negative impact totheir care or other services. Each participant will be sequentially assigned a specific number to maintain confidentiality. All study-related information will be stored securely in a locked file cabinet with an anonymously allocated number and restricted access.

### 3.2. Declaration of Interests and Access to Data

This research is supported by a Science Research Grant (19K20171) from the Japan Society for the Promotion of Science (JSPS) and a research grant from the Japan Society for Lipid Nutrition (2019 Ota Oil Promotion Award). Test foods, blood fatty acid tests, and the gut microbiota tests will be provided by Morinaga Milk Industry Co., Ltd. (Tokyo, Japan), but are not funded by Morinaga Milk Industry Co., Ltd. This study is planned and will be carried out independently by the investigators, and the provision of the above products and goods will not influence the study results in favor of Morinaga Milk Products Co., Ltd. Therefore, Morinaga Milk Industry Co., Ltd. will ultimately have no influence on the research results. The management plan for the investigators’ conflicts of interest in this study has been reviewed by the Institutional Review Board of the National Center of Neurology and Psychiatry and is appropriately managed by the Principal Investigator (RO).

Regarding data access and responsibility, the Principal Investigator (RO) has full access to all of the data in the study and takes responsibility for the integrity of the data and the accuracy of the analysis.

### 3.3. Availability of Data and Materials

The results of this study will be disseminated in presentations at academic conferences and publications in peer-reviewed journals. Eligibility for authorship of these publications will adhere to the authorship criteria of the International Committee of Medical Journal Editors (ICMJE). For transparency of trial results and adherence with ICMJE recommendations [46], individual participant data generated in this study will be anonymized and will be shared with researchers for secondary use of the data upon request in accordance with the data sharing policy described in Table 2.

### 3.4. Patient and Public Involvement

Prior to this study, we conducted a systematic review of cancer survivorship guidelines in Japan and clarified the needs of cancer survivors [47]. In the present study, the research question, study design, and outcome measures were determined based on a discussion with participants of previous studies [18,19,48]. The burden imposed by intervention in this RCT will be assessed by the participants themselves and their opinions will be used in the design of the next RCT.

## 4. Discussion

To our knowledge, this will be the first RCT to explore the effects of nutritional intervention on FCR in cancer survivors. This study will also be the first to examine the effects of a combination of probiotic and omega-3 fatty acids (Bifidobacterium and perilla oil) on psychiatric symptoms. This combination is designed based on our previous observational study and growing interest in interaction between the gut microbiome and omega-3 fatty acids.

This study primarily aims to evaluate the preliminary efficacy and effect size of the study treatment to guide future full-scale confirmatory trials of the effects of Bifidobacterium and perilla oil on FCR in cancer survivors. Due to fewer adverse effects than psychotropic drugs, this nutritional intervention should be acceptable for physically vulnerable cancer survivors. It is hard to imagine that these supplementations alone will replace standard psychotropic drugs and psychotherapy in the future. Rather, they will likely be used to complement other treatments. It is also reasonable to consider them as part of behavior change interventions in daily life (physical activity, weight control, smoking cessation, etc.), with the aim of improving well-being in breast cancer survivors.

The advantage of this treatment over other treatments is that it is easy to implement. Bifidobacterium and perilla oil are commercially available from food manufacturers and are readily available. Psychotropic drugs have side effects, including cognitive impairment which can lead to falls, and the problem with psychotherapy is that there are few psychologists in Japan who can provide treatment. If the effects of Bifidobacterium and perilla oil on FCR in cancer survivors are shown to be effective in future full-scale confirmatory trials, their preventive effects will also need to be examined.

The major limitation of this study is the lack of placebo, without which the intervention group is subject to a placebo effect with the attendant risk of overestimating the effect of the intervention versus the control. However, from our experience, cancer survivors are commonly quite reluctant to participate in studies using a placebo. Additionally, people are cautious about the foods they consume, and most would decline anything other than foods that are nutritious, including wasted calories. Thus, we will not use a placebo capsule or sachet in this pilot study. In the next full-scale confirmatory study, we will use a placebo in the control group. Moreover, many foods contain some form of omega-3 or probiotics. Because this study does not control for diet other than supplementation, it is possible that people may unintentionally consume more than the prescribed amount. This may lead to under- or overestimation of the intervention effect. To address this issue, we will measure blood levels of omega-3 fatty acids at baseline and assess dietary habits using a food frequency questionnaire. In addition, after the intervention, changes in dietary habits, supplementation, and history of antibiotic use will be evaluated through an interview, and a secondary analysis will be conducted to account for these effects and to confirm the robustness of the results.

## Figures and Tables

**Table 1:** Schedule for the assessments.

		Screening Period		Treatment Period	
	Rater	Pre-Entry Screening	Baseline Assessment ^1^	Random Allocation ^1^	Outcome Assessment ^2^
***Enrolment***					
Screening for eligibility	Physician	×			
Informed consent	CRC/Physician	×			
Registration to EDC	CRC		×		
***Assessment***					
Baseline characteristics of participants	CRC		×		
Blood fatty acid test	CRC		×		
Interstitial flora	CRC		×		×
CARS assessed via interview	CRC/masked rater		×(CRC)		×(masked raters)
PHQ-9	PRO questionnaires		×		×
IBS-SI	PRO questionnaires		×		×
Information about allocated groups	CRC			×	
Adverse reactions	CRC/PRO questionnaires				×
				← As needed throughout protocol →	

^1^ Baseline assessment and random allocation for completion within 3 weeks after registration in this study. ^2^ Outcome assessment will be conducted at 12 weeks from random allocation (allowing for a subsequent 14 days).

**Table 2:** Data sharing policy for this study. Abbreviation: IPD, individual participant data.

Data Sharing Policy	
Is IPD available?	Yes
What types of data will be shared?	All anonymized IPD reported in the paper (body text, tables, and figures)
Are there any other documents available?	Protocol paper
How long will the data be available for?	For 5 years after publication of the paper
Who will use the data?	Researchers who have presented plans with appropriate methodologies
What types of analysis will this be used for?	For meta-analyses using IPD
How can the data be accessed?	Interested researchers should send a request for data sharing, along with a synopsis of the secondary analysis plan, to the Principal Investigator (RO) by email (ryo-okubo@ncnp.go.jp). The study administrative office will examine the request and, if approved, send a letter of approval for data sharing to the requesting researcher.

## Data Availability

The results of this study will be disseminated in presentations at academic conferences and publications in peer-reviewed journals. For the transparency of trial results and adherence with ICMJE recommendations, individual participant data generated in this study will be anonymized and will be shared with researchers for secondary use of the data upon request in accordance with the data sharing policy.

## Abbreviations

ARs	adverse reactions
ALA	alpha-linolenic acid
CRC	clinical research coordinator
CRF	case report form
CARS	Concerns about Recurrence Scale
EDC	electronic data capture system
FCR	fear of cancer recurrence
FFQ	Food Frequency Questionnaire
IAP	intestinal alkaline phosphatase
ITT	intention-to-treat
ICMJE	International Committee of Medical Journal Editors
IBS	irritable bowel syndrome
IBS-SI	Irritable bowel syndrome severity index
JRCT	Japan Registry of Clinical Trials
NCNP	National Center for Neurology and Psychiatry Hospital
NHO	National Hospital Organization
PHQ-9	Patient Health Questionnaire-9
PPS	per protocol set
PRO	patient-reported outcomes
PUFA	polyunsaturated fatty acid
